# Composition, Abundance, and Diversity of the Soil Microbiome Associated with the Halophytic Plants *Tamarix aphylla* and *Halopeplis perfoliata* on Jeddah Seacoast, Saudi Arabia

**DOI:** 10.3390/plants12112176

**Published:** 2023-05-30

**Authors:** Naseebh N. Baeshen, Lina Baz, Ashwag Y. Shami, Ruba A. Ashy, Rewaa S. Jalal, Aala A. Abulfaraj, Mohammed Refai, Mazen A. Majeed, Samah S. Abuzahrah, Hayam Abdelkader, Nabih A. Baeshen, Mohammed N. Baeshen

**Affiliations:** 1 Department of Biology, College of Sciences and Arts at Khulais, University of Jeddah, Jeddah 21959, Saudi Arabia; nnbaeshen@uj.edu.sa; 2 Department of Biochemistry, Faculty of Science, King Abdulaziz University, Jeddah 21589, Saudi Arabia; lbaz@kau.edu.sa; 3 Department of Biology, College of Sciences, Princess Nourah bint Abdulrahman University, Riyadh 11617, Saudi Arabia; ayshami@pnu.edu.sa; 4 Department of Biology, College of Science, University of Jeddah, Jeddah 21493, Saudi Arabia; raashy@uj.edu.sa (R.A.A.); rsjalal@uj.edu.sa (R.S.J.); mazen-mj@hotmail.com (M.A.M.); mnbaeshen@uj.edu.sa (M.N.B.); 5 Biological Sciences Department, College of Science & Arts, King Abdulaziz University, Rabigh 21911, Saudi Arabia; aaabulfaraj@kau.edu.sa; 6 Department of Biochemistry, College of Science, University of Jeddah, Jeddah 21493, Saudi Arabia; mrefai@uj.edu.sa; 7 Virus Research Department, Molecular Biology Laboratory, PPRI, ARC, Giza 12613, Egypt; hsabdelkader@uj.edu.sa; 8 Department of Biological Sciences, Faculty of Science, King Abdulaziz University, Jeddah 21589, Saudi Arabia; nabih_baeshen@hotmail.com

**Keywords:** microbial communities, *Tamarix aphylla*, *Halopeplis perfoliata*, 16S rRNA gene, ITS rRNA gene, alpha diversity, beta diversity

## Abstract

The coast of the Red Sea in Jeddah City is home to a unique microbial community that has adapted to extreme environmental conditions. Therefore, it is essential to characterize the microbial community in this unique microbiome to predict how environmental changes will affect it. The aim of this study was to conduct metagenomic sequencing of 16S rRNA and ITS rRNA genes for the taxonomic classification of the microbial community in soil samples associated with the halophytic plants *Tamarix aphylla* and *Halopeplis perfoliata*. Fifteen soil samples were collected in triplicate to enhance robustness and minimize sampling bias. Firstly, to identify novel microbial candidates, the gDNAs were isolated from the saline soil samples surrounding each plant, and then bacterial 16S (V3–V4) and fungal ITS1 regions were sequenced utilizing a high-throughput approach (next-generation sequencing; NGS) on an Illumina MiSeq platform. Quality assessment of the constructed amplicon libraries was conducted using Agilent Bioanalyzer and fluorometric quantification methods. The raw data were processed and analyzed using the Pipeline (Nova Lifetech, Singapore) for bioinformatics analysis. Based on the total number of readings, it was determined that the phylum *Actinobacteriota* was the most prevalent in the soil samples examined, followed by the phylum *Proteobacteria*. Based on ITS rRNA gene analysis, the alpha and beta fungal diversity in the studied soil samples revealed that the fungal population is structured into various groups according to the crust (c) and/or rhizosphere (r) plant parts. Fungal communities in the soil samples indicated that Ascomycota and Basidiomycota were the two most abundant phyla based on the total amount of sequence reads. Secondly, heat-map analysis of the diversity indices showed that the bacterial alpha diversity, as measured by Shannon, Simpson, and InvSimpson, was associated with soil crust (Hc and Tc enclosing *H. perfoliata* and *T. aphylla*, respectively) and that the soil rhizosphere (Hr and Tr) was strongly correlated with bacterial beta diversity. Finally, fungal-associated Tc and Hc samples clustered together, according to observations made using the Fisher and Chao1 methods, and Hr and Tr samples clustered together according to Shannon, Simpson, and InvSimpson analyses. As a result of the soil investigation, potential agents that have been identified could lead to innovative agricultural, medical, and industrial applications.

## 1. Introduction

The soil is home to thousands of different bacterial, archaeal, and eukaryotic taxa, all of which contribute to the soil’s unique biological makeup and, in turn, its unique functions. These microbial communities are highly variable, with either free-living or symbiotic members producing either harmful or helpful outcomes [1,2]. Moreover, plants can change the bacterial and fungal composition of the soil microbiome, creating more favorable conditions, by excreting specific metabolites from their roots, such as benzoxazinoids [3].

More than 80% of plants have associated mycorrhizal fungi, especially in their roots, which makes these fungi a crucial component of the microbiome in plant–soil interactions [2,4]. Moreover, a recent study that compared fungi and bacteria revealed that both had the strongest correlations with plant diversity and microbial richness [1]. Furthermore, deeper soil layers, such as those surrounding plant roots, also significantly mediate these interactions. However, little is known about these layers compared to the rhizosphere [5].

A study of the distribution of fungi in Saudi soil showed high variation in terms of the dominant fungal classes and genera [6]. The dominant classes were Sordariomycetes, Dothideomycetes, Eurotiomycetes, and Eurotiomycetes [7]. The study reported that Eurotiomycetes, Pezizomycetes, and Sordariomycetes were the dominant classes; furthermore, the genus level showed similar variation [7]. The study reported that *Aspergillus*, *Fusarium*, *Myrothecium*, and *Penicillium* were the dominant genera [7], while another reported that *Aspergillus, Madurella,* and *Thielavia* were dominant [6]. *Alternaria*, *Aspergillus*, and *Fusarium* have also been cited as being the most abundant [8]. These studies were carried out without specific reference to the soil type or surroundings in terms of plant growth.

The area around Jeddah is characterized by harsh weather and climatic changes, including biotic and abiotic stress conditions, such as high humidity, drought, precipitation, and high temperatures. Despite this, different areas along the corniche have minimal halophytic plant growth, which explains their survival in stressed environmental conditions [9]. In arid and semi-arid areas, as well as in wetlands with high salinities, *Halopeplis perfoliata* are typically found. These halophytic desert plants can survive in dry environments and generally are associated with symbiotic bacteria that enable them to withstand harsh environmental conditions [10,11,12]. Additionally, *H. perfoliata* has several industrial and ecological advantages [10,11,12].

*Tamarix aphylla* is a desert and halophytic plant that can grow in and tolerate saline and alkaline soils [13]. The plant is widely used in folk medicine in Saudi Arabia, such as in the treatment of fever, skin diseases, wounds, and swellings, and as a diuretic [14]. Numerous metagenomics studies have examined the soil microbial communities in cold and warm deserts, forests, grasslands, and tundra [15,16]. However, few of these studies have examined the soil microbiome in relation to halophytes. Therefore, studying how halophytes interact with soil microbial communities and how these interactions promote growth and survival under abiotic stresses is important. Such interactions attest to the vast array of microorganisms that promote crop plant growth under various biotic and abiotic stresses, which can be used as biological agents in various industrial and medical settings [17,18].

The objectives of this study were to characterize the microbial community associated with halophytic plants on the Red Sea coast in Jeddah, conduct metagenomic sequencing to taxonomically classify the microbial community, assess the prevalence and diversity of bacteria and fungi, evaluate the impact of environmental factors on microbial diversity, and identify potential microbial candidates with applications in agriculture, medicine, and industry.

## 2. Materials and Methods

### 2.1. Sample Collection

This research was conducted near an electricity station on the seacoast of Jeddah’s southern corniche, in Saudi Arabia, at a latitude of 21.2181343° N and a longitude of 39.1749619° E. Firstly, five soil samples were collected on 14 July 2020 at 11:00 h, at a temperature of 38 °C, from areas where *T. aphylla* and *H. perfoliata* grow. Secondly, to ensure robust statistical analysis, triplicate samples were collected first, followed by the combination of all triplicates into a single sample to enhance the quality and accuracy of DNA for NGS (next-generation sequencing). Furthermore, the samples were labeled based on the plant species (H: *H. perfoliata* or T: *T. aphylla*) as the first letter and the type of soil (c: crust or r: rhizosphere) as the second letter. Thus, Hc samples were obtained from the crust soil surrounding *H. perfoliata* plants; Hr samples were obtained from the rhizosphere soil surrounding *H. perfoliata* plants; Tc samples were gathered from crust soil surrounding *T. aphylla* plants; Tr samples were collected from rhizosphere soil surrounding *T. aphylla* plants; and control samples were collected from a nearby area with no plant growth.

### 2.2. PCR Amplification and Next-Generation Sequencing

A commercial FastDNATM Spin Soil Kit (MP Biomedicals, Santa Ana, CA, USA) was used to extract genomic DNA. The iQuantTM Broad Range dsDNA Quantification Kit was used for fluorometric quantification, while the Implen NanoPhotometer^®^ N60/N50, (Implen, Inc., CA 91362, USA) spectrophotometer was used to determine the DNA concentration. The bacterial V3–V4 16S rRNA gene fragments were amplified using PCR with universal primers: Forward: 5′-CCTACGGGNGGCWGCAG-3′ and Reverse: 5′-GACTACHVGGGTATCTAATCC-3′ [19]. In addition, the fungal ITS1 region of the rRNA gene sequence fragments was amplified by PCR with the primers ITS5 (5′-GGAAGTAAAAGTCGTAACAAGG-3′) and ITS2 (5′-GCTGCGTTCTTCATCGATGC-3′) [15]. The PCR amplification was performed using an initial denaturation at 94 °C for 5 min, followed by 30 cycles of denaturation at 94 °C for 30 s, annealing at 57 °C for 40 s, and extension at 72 °C for 1.30 s, with a final elongation at 72 °C for 10 min [20]. Purified amplicons were used for library construction and deep sequencing on Illumina SBS technology to recover 300 bp pair-end reads of the V3 and V4 regions [20]. Following the Illumina-recommended methodology, pooled and normalized purified amplicons were used for the construction of libraries for sequencing. The MiSeq platform (300 PE) was used for the sequencing process. For the genotyping of fungal taxonomy and diversity, 18S rRNA, ITS1, and ITS2 are the most frequently used variable; ITS is more suitable as a genetic marker used to measure genetic diversity within a species, and produces products with sizes appropriate for short reads in Illumina sequencing platforms [21].

### 2.3. Statistical Analysis

Utilizing the BBDuk tool from the BBTools package, sequence cleanup was carried out by eliminating low-quality and paired-end reads (https://sourceforge.net/projects/bbmap), accessed 10 September 2022. Then, using USEARCH v11.0.667, the forward and reverse reads were combined (https://www.drive5.com/usearch), accessed 10 September 2022. Following these quality checks, reads were clustered into operational taxonomic units (OTUs) using UPARSE v11.0.667 based on their 97% similarity. QIIME V1.9.1 (http://qiime.org), Accessed 10 September 2022. used the Ribosomal Database Project (RDP) Classifier to determine the phylogenetic pattern of its input data (http://qiime.org/), accessed 10 September 2022. To determine diversity and richness, alpha-diversity was evaluated using Shannon, InvSimpson, Simpson, Fisher, and Chao1 indices in the Mothur software package (http://www.mothur.org), accessed 10 September 2022. A rarefaction curve was created by determining the OTU numbers of the extracted tags and the greatest depth permitted to preserve all samples. Beta diversity was assessed using statistical techniques such as principal coordinate analysis (PCoA), redundancy analysis (RDA), non-metric multidimensional scaling (NMDS), canonical correspondence analysis (CCA), and multidimensional scaling (MDS). These methods leveraged the evolutionary information within the system to compare species composition across samples and determine the variation in community structure. Finally, PCoA was performed using weighted and unweighted UniFrac distance metrics to determine the similarity between different soil samples and classify the various soil types [15,22]. The online calculator VENNY v2.1 was used to create the Venn diagram (https://bioinfogp.cnb.csic.es/tools/venny/), accessed 10 September 2022.

## 3. Results

### 3.1. 16S rRNA Sequence Assembly and the Total Number of OTU Reads

Metagenomics techniques were used to explore the diversity of the soil bacteria in the *T. aphylla* and *H. perfoliata* samples. The results of the assembly and the total number of sequence reads for the samples are shown in Table 1. Bacterial OTUs were highest, at 1559, in the *T. aphylla* rhizosphere sample; meanwhile, the lowest value was 120, which was found in the control sample.

### 3.2. ITS rRNA Sequence Assembly and the Total Number of OTU Reads

Metagenomics methods were used to explore the association between the diverse microbial communities of eukaryotic organisms and the *T. aphylla* and *H. perfoliata* soil samples. The results of the assembly and the overall number of fungal sequence reads across the samples are shown in Table 2. Clustered samples were assigned to the OTUs, which are presented in Table 2. The fungal OTU reached its highest value at 184, which was associated with the *T. aphylla* crust samples. In contrast, the lowest value was 87 OTUs, which was related to the *H. perfoliata* rhizosphere samples.

### 3.3. 16S rRNA Taxonomic Classification at the Phylum and Genus Levels

**Figure 1:**
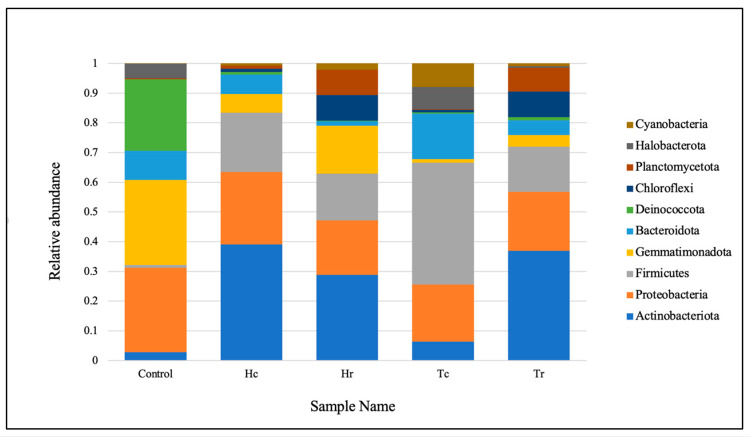
The abundance rates of the dominant phyla of bacterial communities in the soil samples. Control: control sample; (Hc): *H. perfoliata* crust sample; (Hr): *H. perfoliata* rhizosphere sample; (Tc): *T. aphylla* crust sample; (Tr): *T. aphylla* rhizosphere sample.

The phylum-level taxonomic distribution of the microbial communities in the samples revealed that *Actinobacteriota* were the most highly represented at the phylum level, based on the entire sequence reads. *Proteobacteria* was the second most common phylum, followed by *Firmicutes, Gemmatimonadota*, *Bacteroidota*, *Deinococcota*, *Chloroflexi*, *Planctomycetota*, *Halobacterota*, and *Cyanobacteria* (Figure 1). The top ten bacterial communities in terms of class composition were *Actinobacteria*, *Bacilli*, *Alphaproteobacteria*, *Gammaproteobacteria*, *Longimicrobia*, *Bacteroidia*, *Deinococci*, *Acidimicrobiia*, *Planctomycetes*, and *Halobacteria* (see Figure S1). Meanwhile, for the order level, the top ten in terms of taxonomic distribution were *Bacillales*, *Longimicrobiales*, *Frankiales*, *Deinococcales*, *Propionibacteriales*, *Cytophagales*, *Burkholderiales*, *Rhizobiales*, *Caulobacterales*, and *Halobacterales* (see Figure S2). The top taxonomic distribution for the family composition was *Bacillaceae, Longimicrobiaceae*, *Nocardioidaceae*, *Trueperaceae*, *Geodermatophilaceae*, *Hymenobacteraceae*, *Hyphomonadaceae*, *Rhodobacteraceae*, *Beijerinckiaceae*, and *Halomonadaceae* (see Figure S3).

**Figure 2:**
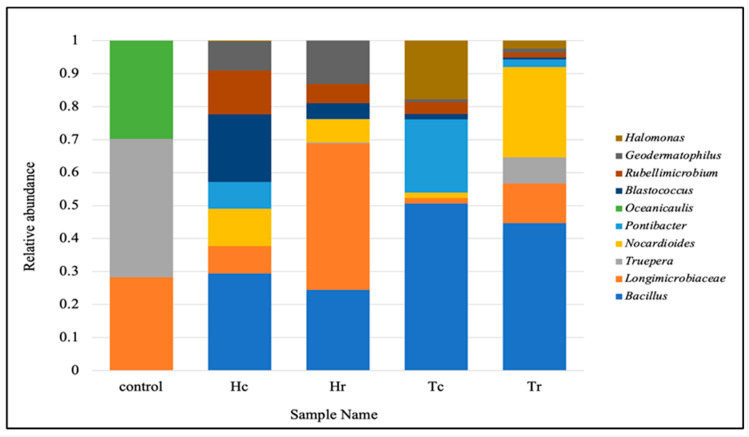
The abundance rates of the dominant genera of bacterial communities for the soil samples.

Based on all of the sequence reads, the top ten taxonomic distributions at the genus level revealed that *Bacillus* was the most prevalent, followed by *Longimicrobiaceae*, *Truepera*, *Nocardioides*, *Pontibacter*, *Oceanicaulis*, *Blastococcus*, *Rubellimicrobium*, *Geodermatophilus*, and *Halomonas* (Figure 2). The top ten phyla are listed on the right side with a color key, along with the abundance rates of the dominant phyla in the five soil samples’ bacterial communities (Figure 2).

### 3.4. ITS rRNA Gene-Based Taxonomic Classification and the Relative Abundance of the Fungal Community

**Figure 3:**
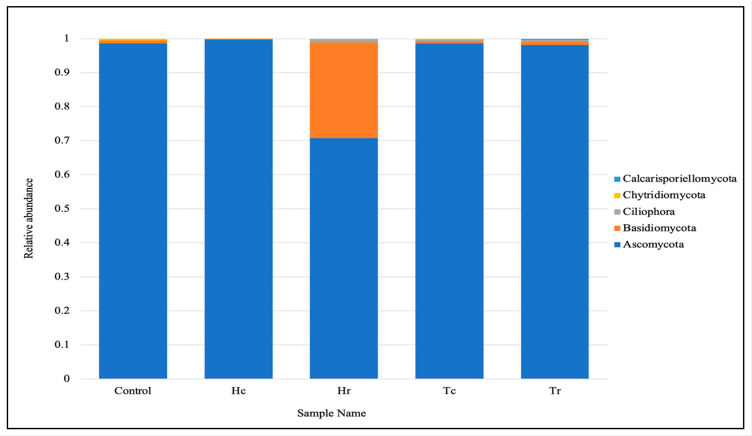
Fungal communities within each sample are based on their respective phyla.

Based on the total readings, the fungal microbial communities in the samples were found to belong to five phyla, according to the taxonomic distributions at the phylum level. The most common phylum was Ascomycota; based on the total readings, Basidiomycota was the second most common phylum, followed by Ciliophora, Chytridiomycota, and Calcarisporiellomycota (Figure 3). The top eukaryotic microbial communities in the class composition of the soil samples were Sordariomycetes, Eurotiomycetes, Dothideomycetes, Agaricomycetes, Spirotrichea, Rhizophlyctidomycetes, Pezizomycetes, Calcarisporiellomycetes, Oligohymenophorea, and Malasseziomycetes (see Figure S4). Based on the total readings, the top orders of the taxonomic distribution were Eurotiales and Sordariales, followed by Dothideales, Capnodiales, Cantharellales, Hypocreales, Xylariales, Pleosporales, Sporadotrichida, and Polyporales (see Figure S5). The top eukaryotic microbial communities in the family composition of the soil samples were Aspergillaceae, which were the most widespread in the taxonomic distribution. They were followed by Dothideales_fam_Incertae_sedis, Teratosphaeriaceae, Ceratobasidiaceae, Pleosporaceae, Sordariaceae, Rhizophlyctidaceae, Chaetomiaceae, Ganodermataceae, and Cordycipitaceae (see Figure S6).

**Figure 4:**
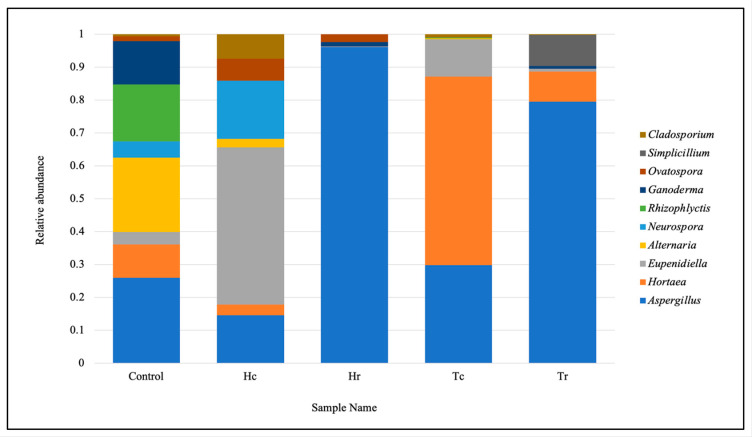
Fungal communities within each sample are based on their respective genera.

Based on the total reads, analysis of the top ten taxonomic groups at the genus level revealed that *Aspergillus* was most abundant, followed by *Hortaea*, *Eupenidiella*, *Alternaria*, *Neurospora*, *Rhizophlyctis*, *Ganoderma*, *Ovatospora*, *Simplicillium*, and *Cladosporium* (Figure 4).

### 3.5. 16S rRNA Community Richness and Diversity (Alpha and Beta)

**Figure 5:**
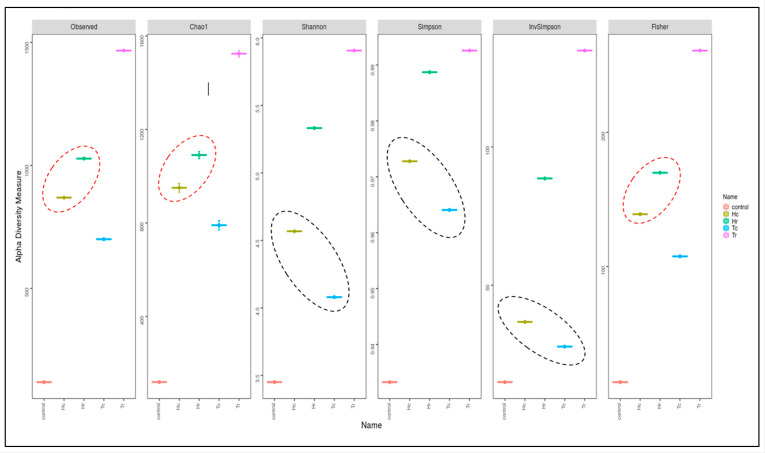
Cluster analysis of the alpha diversity indices for the examined soil samples. The red dotted circles show the intensity of biodiversity convergence between the two regions HC&HR. The black area circles also indicate the intensity of the biodiversity convergence of the two regions HC&T.

**Figure 6:**
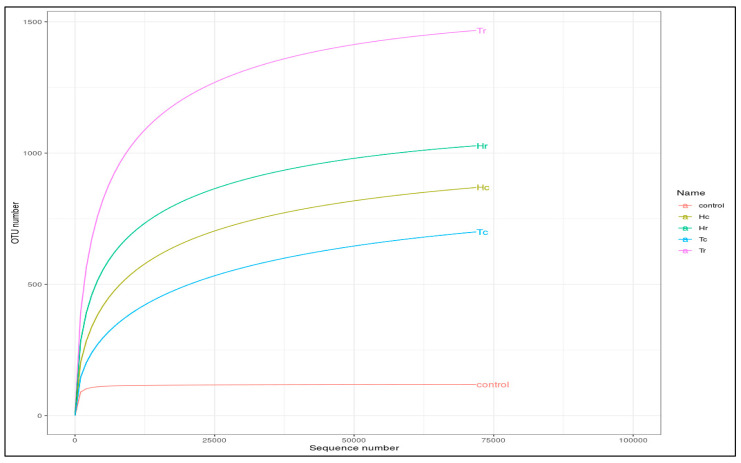
An illustration of the species richness of five soil samples using alpha rarefaction curves.

Assessed based on a number of indices, alpha diversity was used to analyze the species complexity (Chao1, Shannon, Simpson, inverse Simpson, and Fisher). Beta diversity is essential for understanding the relationship between species diversity and habitats. Figure 5 presents cluster analysis of alpha diversity, compared across the samples. Tr demonstrated the highest diversity and richness, according to all diversity indices. Nevertheless, other indices produced different rankings for other samples. For example, various outcomes were achieved for Hr, Hc, and Tc in the alpha diversity index. While Hr ranked second in OTUs, Hc and Tc had comparable OTUs in the alpha diversity indices to the control. The alpha rarefaction curve indicates whether the analysis read count is sufficient for detecting species/OTUs. The curve flattened out to the right, suggesting that the analysis employed a sufficient number of reads (Figure 6). Tr is rated first in terms of species richness, having the greatest concentration of species, a number of different species, and an abundance of species. Moreover, it had the most OTU variations across all metrics.

The rarefaction curves for the observed species richness (Figure 6) came close to saturation. In keeping with the theory that observed species richness is often associated with total OTU richness, Tr displayed the highest species richness while Tc had the fewest OTUs. The sites with the next highest OTU richness were Hr, Hc, and Tc,, respectively, according to the rarefaction curves.

**Figure 7:**
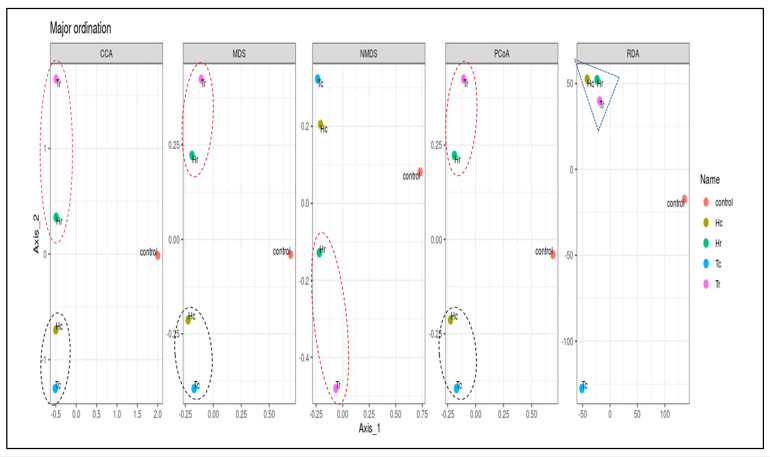
Box plots of beta-diversity indices comparing microbial community diversity between the samples. The beta diversity index (RDA) represented by the triangle figure showed for the first time the convergence between the three samples (Hr, Tr, Hc) while the circular figure did not show this correlation.

The bacterial alpha diversity expressed as the Shannon, Simpson, and InvSimpson diversity of 16S rRNA genes of the Hc and Tc samples was significantly correlated with the plant part (crust). In contrast, the alpha diversity, expressed in terms of Observed, Fisher, and Chao1 analysis, in the Hr and Hc soil samples was correlated with plant species (*H. perfoliata*). Furthermore, the bacterial beta diversity, expressed by the CCA, MDS, NMDS, and PCoA of 16S rRNA genes of the Hr and Tr samples, which were clustered together (Figure 7), significantly correlated with the plant part (rhizosphere); additionally, the samples Hc and Tc were associated with the plant part (crust) (Figure 5).

**Figure 8:**
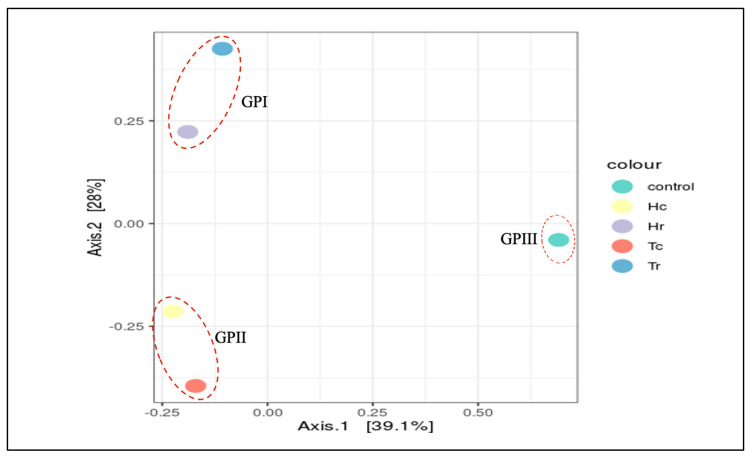
Beta diversity ordination analysis (PCoA) for the samples. Beta diversity analysis of PCoA plot of the the samples. 3 groups are formed according to the species composition; group I: (Hr, Tr), group II: (Hc, Tc), group III: (control).

Several techniques (PCoA, RDA, CCA, MDS, and NMDS) were used for beta diversity analysis, which demonstrates the diversity of and variations in the OTU composition within the samples. The control sample (C) was frequently clustered apart, whereas the rest were clustered differently according to the analysis methods used (Figure 7 and Figure 8).

PCoA was used to study the beta diversity of microbial communities as measured by the 16S rDNA V3–V4 region, and it generated the most accurate clusters. A PCoA plot of the microbial communities (Figure 8) was used to depict data similarities and clustering tendencies. The first and second PCoA axes revealed 28% and 39.1% of the overall variation in the microbial communities. In addition, the PCoA plot indicated that the five soil samples were distributed into three different cluster groups in terms of species composition: group GPI—Tr and Hr, group GPII—Hc and Tc, and group GP III—control (Figure 8).

### 3.6. ITS-rRNA Fungal Community Richness and (Alpha/Beta) Diversity

**Figure 9:**
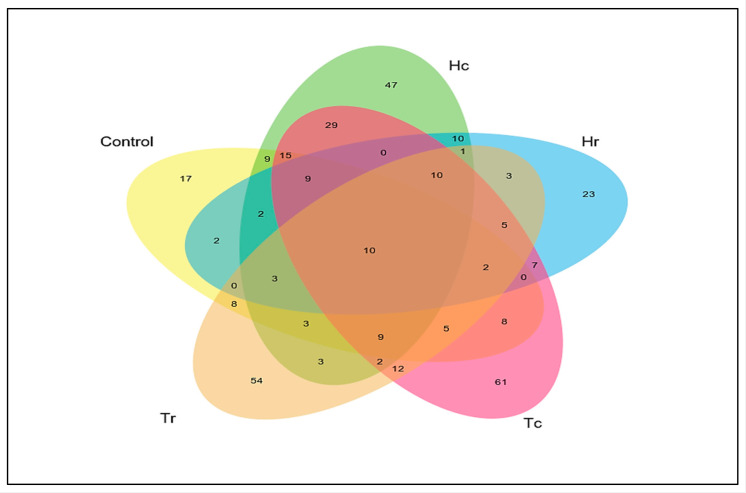
Venn diagram of the shared unique OTUs of the fungal taxa among the soil samples. Venn diagrams showing the numbers of shared and unique OTUs among the experimental plots in the the sample.

The relationships, overlaps, and differences in fungal community richness among the studied soil samples were investigated. The number of distinct and shared OTUs for the ITS-rRNA gene of the fungal sequencing data from the five soil samples are represented in a Venn diagram. The data matrix used to generate this Venn diagram was the presence/absence of all OTUs in the five respective samples (Hc, Hr, Tc, Tr, and the control). Therefore, shared fungal taxa were present in the five soil samples; 10 were operationally defined as the core microbiome (Figure 9).

**Figure 10:**
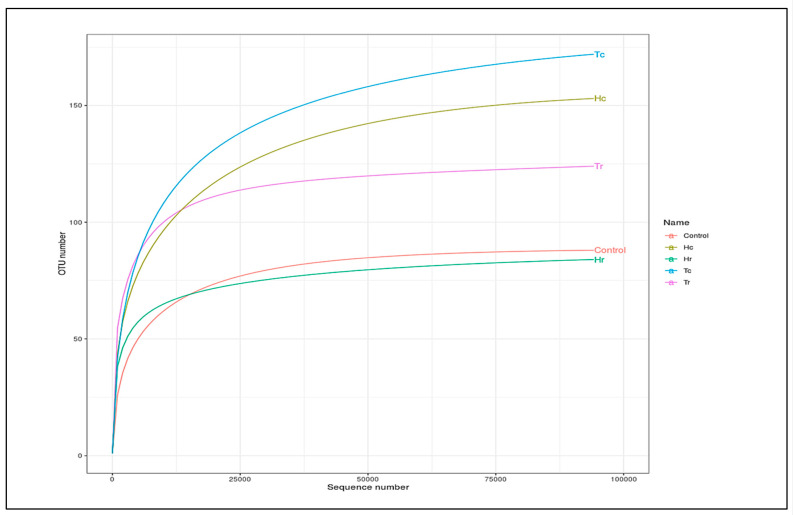
Alpha rarefaction curves demonstrating studied species richness in the samples. Rarefaction curves showing observed species richness in the samples, the IDs of the samples are on the right side.

Enough reads were used when all the rarefaction curves of the observed fungal species richness flattened to the right (Figure 10). The rarefaction curves highlight the differences, overlaps, and correlations between the analyzed soil samples (Hc, Hr, Tc, and Tr) and the control sample. For instance, it was discovered that significantly fewer fungal OTUs were found in the Hr sample than in the control sample (Figure 10).

**Figure 11:**
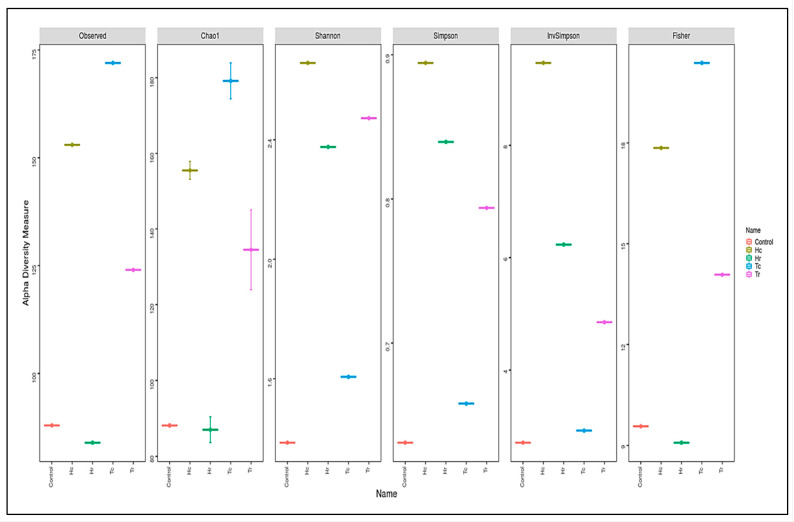
Cluster analysis of alpha-diversity indices comparing fungal community diversity depending on the Simpson, observed Shannon, Fisher, inverse Simpson, and Chao1 values among the samples.

Fungal diversity and richness are represented by alpha diversity indices using the Shannon, Chao1, ACE, Fisher, Simpson, and inverse Simpson indices of the studied samples. The ordering of the additional samples differed across many metrics and revealed various outcomes in terms of the diversity indices (Figure 11).

The sample with the highest richness and diversity was Tc, according to the Observed, Chao1, and Fisher diversity indices (Figure 11). Although Hc ranked second in the Chao1, Fisher, and observed indexes, it had a higher OTU than Tc in the Simpson, Shannon, and inverse Simpson indices. Tc, which was rated first in terms of the abundance of species, species richness, the concentration of species, and the greatest number of different species, had the greatest OTU variances across all metrics. T and Hc are clustered together, according to the observed, Fisher, and Chao1 indices, while Hr and Tr were clustered together according to Shannon, Simpson, and InvSimpson (Figure 11); as such, the samples are clustered based on plant parts (c and/or r). However, the control sample was clustered either with Hr, according to the observed, Fisher, and Chao1 indices, or with Tc, according to Shannon, Simpson, and InvSimpson. Therefore, the control sample might be clustered based on the plant species (*H. perfoliata* or *T. aphylla*) or the plant part (c/r).

**Figure 12:**
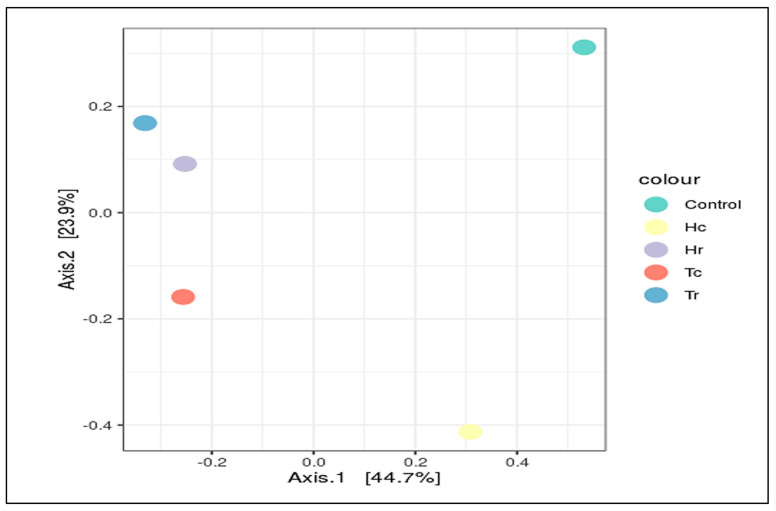
Principal coordinates analysis (PCoA) of fungal community diversity in the samples.

Several analyses (CCA, RDA, NMDS, MDS, and PCoA) were performed for beta diversity to illustrate the variations in OTU composition and fungal diversity across the samples. According to beta diversity, including PCoA and MDS, the control sample (C) was often grouped independently from Tr and Hr. The Tc and Hc groups were geographically and statistically clustered based on the plant part (c/r) and the makeup of the OTUs in each soil sample (Figure S7). All fungal community structures in the studied samples significantly varied in terms of species composition based on the plant part (c/r). The results shown in Figure S7 indicate that Hr and Tr are clustered close to each other according to the PCoA, RDA, CCA, and MDS methods. In contrast, Hc and Tc are clustered close to each other according to the CCA, MDS, and PCoA methods based on the plant part (c/r). In contrast, the control sample is separate regardless of plant species (H/T) or plant part (c/r) (Figure 12). According to the results in Figure S7, Hr and Tr are grouped close to each other using the PCoA, RDA, CCA, and MDS techniques. Hc and Tc, on the other hand, are clustered close to each other using the CCA, MDS, and PCoA algorithms based on plant part (c/r). The control sample, on the other hand, is distinct independent of plant species (H/T) or plant part (c/r) (Figure 12).

### 3.7. Taxonomic Classification and Community Structure of the Soil Microbiome

#### 3.7.1. 16S rRNA Heat-Map Analysis

**Figure 13:**
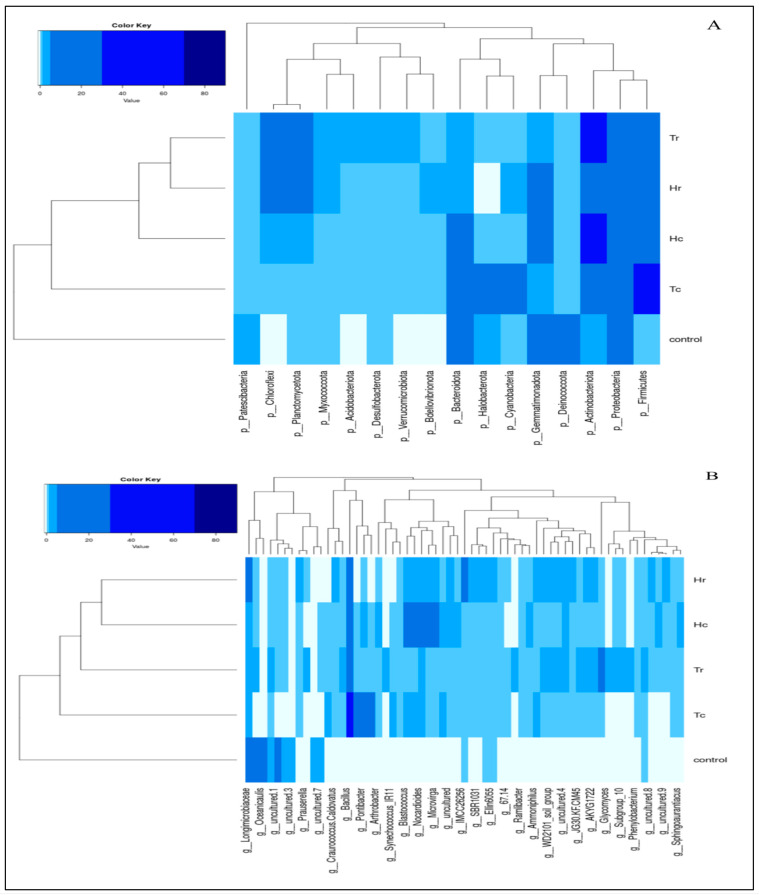
Heat maps and clustering of bacterial soil samples based on 16S rDNA sequence analysis, illustrating the distribution of phyla (**A**) and genera (**B**).

Based on 16S rDNA sequence analysis, *Actinobacteriota* was the dominant phylum among the Gram-positive bacteria, accounting for 39% of the Hc sample, 37% of the Tr sample, and 29% of the Hr sample. In comparison, the control sample had the lowest number (3%) (Figure 13A).

*Proteobacteria* were the most common phylum of Gram-negative bacteria, containing several dangerous genera, including *Escherichia*, *Salmonella*, *Vibrio*, *Yersinia*, and *Legionella*. The results revealed that the control sample included 28% of the phylum *Proteobacteria*, followed by 24% in the Hc sample, 20% in the Tr sample, 19% in the Tc sample, and 18% in the Hr sample.

The phylum *Firmicutes* is a diverse group of spore-forming and non-spore-forming genera of Gram-positive bacteria. *Firmicutes* were found to be the most prevalent phylum (41%) in the Tc sample; 20% were found in the Hc sample, 15% in the Tr sample, and 1% in the control sample.

Tr and Hr had a degree of similarity close to 70%, while Hc clustered with the group that included both Tr and Hr, with about 50% similarity (Figure 13A). The Tr and Hr soil samples were clustered together because of the high percentages of the top ten phyla present in the rhizospheres of both soil samples. Tc was assigned to a distinct and unique clade, but it was clustered together with the Tr and Hr groups, with a similarity of ~5%. The control sample was clustered in a separate clade and had the lowest percentage (0%) of similarity with all other soil samples. The grouping pattern changes as the different classification levels (phylum, class, etc.) change.

At the genus level, the heat map (Figure 13B) revealed that *Bacillus* was the most frequent genus of Gram-positive bacteria in the Tc sample (36%) but represented only 14% of the Hc sample. The Hr sample consisted of 14% *Longimicrobiaceae* and 8% *Bacillus*. Based on plant species, the Hc and Hr cluster groups had a degree of similarity of 25%, whereas Tr was allocated to a separate clade but grouped with the Hc and Hr cluster groups, with a degree of similarity of 18%. Tc was allocated to a separate, unique clade, but was grouped with Hc, Hr, and Tr, with a similarity of less than 5%. The control sample was grouped in a distinct clade and shared the lowest degree of similarity (0%) with all other soil samples.

#### 3.7.2. ITS-rRNA Heat-Map Analysis

**Figure 14:**
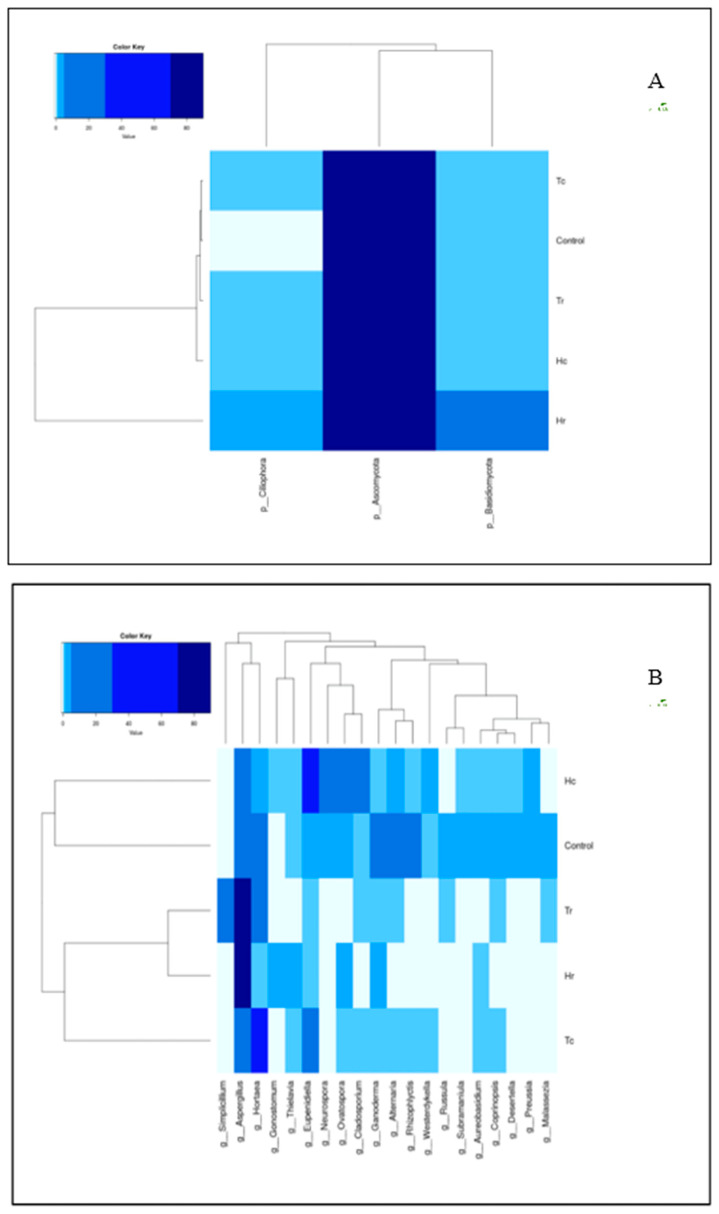
Heat maps and clustering of fungal soil samples based on (ITS) region of the ribosomal RNA (rRNA) gene, illustrating the distribution of phyla (**A**) and genera (**B**).

For the species-level molecular identification of fungi, the internal transcribed spacer (ITS) region of the ribosomal RNA (rRNA) gene was utilized. A two-way hierarchical clustering analysis heat map of communities of micro-eukaryotic species in the samples is shown in Figure 14A,B. According to the color key, the phylum Ascomycota was the most prevalent across all soil samples. In contrast, the phyla Basidiomycota and Ciliophora were only dominant in the Hr sample. There was also an unexpected lack of clustering among the Tc, Tr, Hc, and Hr samples; the Tc, Tr, Hc, and control groups had approximately the same percentage of similarity in terms of the phylum Basidiomycota, whereas the Tc, Tr, and Hc groups shared the same percentage of similarity for the phylum Ciliophora. In addition, the clustering of the control group was closer to the plant species (*T. aphylla*) than to the plant part (c/r).

The relative similarity between soil samples is indicated by the branch lengths in the dendrograms generated by the cluster analysis. All of the samples’ genus composition percentages are represented by a color gradient from deep blue (highest) to light blue (lowest). There was an association between all of the genera (columns) and the soil samples (rows). (Figure 14B). However, the genus composition of each sample varied significantly.

The Tr and Hr samples demonstrated an increased degree of similarity, close to 75%, while the Hc and control groups were clustered together in their clade and had the lowest percentage of similarity. Tc was assigned to a distinct and unique clade, but it clustered with a group that included both Tr and Hr, with less than 10% similarity. Tr and Hr were clustered together, since a high percentage (~75%) of the dominant genus, *Aspergillus*, was identified in the rhizospheres of both samples. The cluster pattern changes as one moves up through the different classification levels (phyla, class, etc.).

## 4. Discussion

Over the past ten years, research on soil microbiome diversity in saline soil and the hot desert environment has expanded due to global climate change, with dry places considered particularly vulnerable [23]. Therefore, the purpose of the present study was to determine whether there was a significant relationship between the soil microbial diversity and *T. aphylla* and *H. perfoliata* plants, and, if there was such a relationship, which factors were responsible for establishing these interactions. The study was conducted near an electricity station on the seacoast of Jeddah, Saudi Arabia. Furthermore, we sought to determine the isolated strains and biodiversity in the studied area on the basis that the ability of organisms to survive in the presence of different environmental stresses may have an impact on the adaptation of this viable biodiversity. It can enable the identification of beneficial strains that may have many agricultural, economic, and industrial implications.

Due to the environmental requirements, nutritional needs, and media compositions of soil microbial communities, the conventional microbial-cultivation-based isolation approaches are inefficient for this kind of research [9]. Although modern techniques could exploit this, the results of studying the soil microbiome diversity, including sample collections, NGS, bioinformatic analysis, and data processing, revealed the structural composition of the microbiota populations related to *T. aphylla* and *H. perfoliata*. Five soil samples were collected from areas where *T. aphylla* and *H. perfoliata* were grown. The Hc samples were obtained from crust soil around *H. perfoliata*; the Hr samples were gathered from rhizosphere soil around *H. perfoliata*; the Tc samples were gathered from crust soil around *T. aphylla*; and the Tr samples were collected from rhizosphere soil around *T. aphylla*. Finally, the control samples were collected from an unvegetated area.

Under these harsh conditions, the ITS1 region of the fungal rRNA gene exhibited fungal biodiversity, while the V3–V4 sections of the bacterial 16S rRNA gene revealed bacterial biodiversity. The differences between the bacterial combinations were investigated in the five soil samples, and high-quality sequences were collected and categorized at the phylum and genus levels. Moreover, bacterial diversity and richness were investigated, and it was revealed that the samples varied significantly according to the number of OTUs.

The sequencing results showed that the bacterial communities were taxonomically distributed into ten phyla at the phylum level. The phylum-level taxonomic distribution shows that the phylum *Actinobacteriota* is the most abundant among the Gram-positive bacteria (Figure 1). The literature states that these bacteria have a wide range of ecological and environmental advantages. *Actinobacteria*, which are Gram-positive filamentous bacteria, have an increased DNA content of cytosine and guanine [24]. *Actinobacteria* carry out a number of crucial functions, such as the breakdown of all varieties of organic molecules. These biosynthetic gene clusters are abundant in these filamentous bacteria, leading to their unrivaled ability to produce diverse bioactive secondary metabolites [25]. Marine *Actinobacteria* are recognized as a ‘treasure trove’ of secondary metabolites due to their ability to create novel bioactive compounds. The bacterial family *Actinomycetaceae* produces most of the bioactive metabolites, including the genera *Acanobacterium*, *Actinobaculum*, and *Streptomyces* [26]. It is believed that each strain of *Actinobacteria* has the genetic potential to produce 15–25 secondary metabolites. *Actinobacteria* have produced over 10,000 antibiotics, or 45% of all identified bioactive microbial compounds [27]. Several research groups were encouraged to continue their work on *Actinobacteria* by the development of the modern approach to recovering marine microorganisms from various environment samples and conducting bioactivity screening. In addition to seawater and sediments, *Actinobacteria* are abundant in marine environments, such as sponges, fish, mollusks, mangroves, and seaweeds [24,28]. These organisms are essential from taxonomic and ecological perspectives, as well as for the development of novel bioactive molecules, made possible by their distinctive carbon skeletons, which contain antioxidants, cytotoxic, antitumor, antibiotic, and cardiovascular agents, and immunosuppressants, and which therefore serve as a solid basis to develop treatments [27].

In the current study, *Proteobacteria* were revealed to be the second most vital phylum, according to the total readings (Figure 1), followed by the remaining phyla in descending order of abundance: *Firmicutes*, *Gemmatimonadota*, *Bacteroidota*, *Deinococcota*, *Chloroflexi*, *Planctomycetota*, *Halobacterota*, and *Cyanobacteria*. *Proteobacteria* are abundant and diverse in several of Earth’s biomes. *Proteobacteria* influence several pathways, including denitrification, autotrophy, hydrogen and sulfur oxidation, and sulfate reduction, in productive marine environments such as deep-sea hydrothermal systems [16]. Aside from chemoautotrophy, hydrothermal *Proteobacteria* lineages are poorly studied. Moreover, *Roseobacter* and *Proteobacteria* are abundant heterotrophic bacterioplankton lineages in surface oceans and are particularly essential in the organic sulfur cycle [16,29]. Several studies have identified *Proteobacteria* as a potential microbial signature in metabolic disorders and inflammatory bowel disease [26]. *Proteobacteria* are ideal for studying genome diversification and environmental adaptation due to their marine abundance and diverse metabolic traits [29,30].

The fast-germinating spores of *Firmicutes* render them widespread in aquatic ecosystems, and surface adhesion might be a survival and evolution strategy in lakes [31]. *Firmicutes* play a significant role in biodegradation processes, such as those involving polychlorinated biphenyls (PCBs) and hexahydro-1,3,5-trinitro-1,3,5-triazine-contaminated soil [29]. *Firmicutes* are sulfate-reduction precursors and can anaerobically decompose organic compounds found within aquatic ecosystems [32].

Several large-scale studies have emphasized the importance of topsoil (0–20 cm) microbial communities. In extreme ecosystems, most plant roots are found within the subsoil (over 20 cm deep), which contains 35% of the microbial biomass. Subsoil microbial reservoirs are vital for complex organic decomposition and the development of the soil structure. Our understanding of the relationships between the subsurface environment and microbial communities has been improved by advances in sequencing technologies [17,18].

High-quality fungal sequences were retrieved and identified at the phylum and genus levels in each soil sample. Each sample was analyzed to measure the diversity and richness of the fungal community, and a broad range of fungi was revealed. Fungal communities in the soil samples showed that Ascomycota and Basidiomycota (Figure 3) were the two most abundant phyla based on the total number of sequences read [18]. This suggests that these phyla influence marine environments [21,22,23].

Marine ascomycetes are a crucial biological grouping of predominantly saprophytic fungi found on a wide variety of substrata that are rich in lignin, cellulose, and chitin [33]. The capacity of these fungi to break down lignocellulose is used on other trophic levels, allowing this complex substrate to enter the food chain [33]. Fungi from the phylum Ascomycota are commonly found in the topsoil of arid and salt-prone grasslands [34]. Ascomycota are significant regulators of the carbon–nitrogen cycle and plant interactions [35], in contrast to Basidiomycota, which dominate the fungal biomass in forest soils rich in organic matter. However, little is known about the roles of Basidiomycota in forest soils where organic matter, water, and nutrients are scarce or only occasionally present. Basidiomycetes are also predominantly saprophytes [22], but are typically excluded from aquatic environments [33], resulting in lower abundances. Moreover, numerous studies have demonstrated the environmental and ecological benefits of these phyla [15,16].

It is believed that fungi play a part in stabilizing soils against seasonal plant biomass decomposition and erosion, as well as direct interaction with plants as either internal cells or as pathogens that stimulate disassembly of selective plant tissues. The results reported by Challacombe et al. (2019) revealed that these fungi play a vital role in cyanobacterial-dominated biological soil crusts and subterranean microhabitats, in which they might aid in the transport of nutrients that act like mycorrhizal fungi, boosting plant survival and growth and enhancing biocrust stability [35].

The data obtained in this study are consistent with the major trends found in research on soil [36,37], marine environments [38,39], mangroves [33,40], and in general, microhabitats [37,41]; all indicate the predominance of Ascomycota and Basidiomycota [42].

This research advances our knowledge of the various ecological roles that are performed by these fungi through the characterization of their genomic features. We conclude that at least some of the isolates interact with plants, and our findings are consistent with this hypothesis. Furthermore, many of the fungi likely display enhanced ecological plasticity; as a result, they can take on various roles based on fluctuating environmental conditions or the growth substrate.

## 5. Conclusions

This research demonstrates the richness of the microbial communities related to stress-tolerant halophytic plants within the soil of Jeddah’s southern corniche. The Red Sea, which is renowned for its ecological diversity and is distinguished by unusual surroundings, is reached after passing through the severe desert of the Arabian Peninsula, as well as salty regions and saline marshes. Therefore, it is necessary to conduct further research into the microbial communities that are stifling this ecosystem. Our findings show that rhizospheric bacteria could serve as biomarkers of plant growth-promoting rates and resilience to abiotic stressors. Hence, this study enables the identification of new candidates with the potential to be employed as biological agents to improve commercial and agricultural processes. In addition to this identification of soil bacterial communities using high-throughput molecular tools for the classification of the taxonomic and phylogenetic relationships, more thorough illustrations and comparative functional and biochemical research into the variability of the soil microbiome are required in order to shed light on various metabolic mechanisms. In the future, researchers must seek to identify new candidates that have the potential to improve humanity’s ecological situation and its resource management; this can be achieved by combining the taxonomic composition and functional traits of the soil microbiome. Additionally, these findings can advance our knowledge of microbial community formation, maintenance, and diversity, offering a new perspective on the recovery of areas that have suffered ecological damage and addressing desertification using particular microbial taxa.

## Figures and Tables

**Table 1:** Outcomes of bacterial sequence gathering for the samples.

Sample Name	Total Reads ^1^	OTUs	Q20 (%)	Q30 (%)	GC (%)
Control-16S	27204681	120	95.79	87.83	58
Hc-16S	67619892	858	93.51	85.88	58
Hr-16S	60276009	986	93.02	84.97	59
Tc-16S	61078118	1120	94.19	86.83	57
Tr-16S	56330547	1559	92.96	84.78	58

^1^ Total Reads: the total number of bacterial sequences read. OTU: classified a unique sequence set with a cutoff of 97% identity. Q20 (%): the proportion of bases where the Phred score is higher than 20. Q30 (%): the proportion of bases where the Phred score exceeds 30. GC (%): GC content of the sequence reads.

**Table 2:** Results of the five soil samples: ITS rRNA sequence assembly.

Sample Name	Total Reads (Mb)	OTUs	Q20 (%)	Q30 (%)	GC (%)
Control—ITS	34119109	102	97.31	92.28	48
Hc—ITS	39738492	162	97	92.65	50
Hr—ITS	45080296	87	95.71	90.08	49
Tc—ITS	47581365	184	96.56	91.76	54
Tr—ITS	48912745	130	96.01	90.18	53

Total Reads: The total number of bases in reads identified. OTUs: Operational taxonomic unit (OTU) analysis. GC (%): the GC percentage in sequence reads. Q20 (%): the percentage of bases in which the phred score is above 20. Q30 (%): The percentage of bases in which the phred score is above 30.

## Data Availability

Not applicable.

## Supplementary Materials

The following supporting information can be downloaded at: https://www.mdpi.com/2223-7747/12/11/2176/s1, Figure S1: The abundance rates of the most prevalent class of bacterial communities in soil samples; Figure S2: The abundance rates of the most prevalent order of bacterial communities in soil samples; Figure S3: The abundance rates of dominant family of bacterial communities among the soil samples; Figure S4: Fungal communities within each sample based on respective class; Figure S5: Fungal communities within each sample based on respective order; Figure S6: Fungal communities within each sample based on respective family; Figure S7: Box plots of beta diversity indices of fungal community richness and composition according to CCA, MDS, NMDS, PCoA, and RDA analysis methods among the samples. Control: Control sample; (Hc): *H. perfoliata* crust sample; (Hr): *H. perfoliata* rhizosphere sample; (Tc): *T. aphylla* crust sample; (Tr): *T. aphylla* rhizosphere sample.
