# Supplementary material for: Composition, Abundance, and Diversity of the Soil Microbiome Associated with the Halophytic Plants Tamarix aphylla and Halopeplis perfoliata on Jeddah Seacoast, Saudi Arabia
Source: Plants (Basel). 2023 May 30;12(11):2176. doi: 10.3390/plants12112176 (PMC10255354; doi:10.3390/plants12112176)
Supplement: Supplementary file 1 [file plants-12-02176-s001.zip › plants-2365659-supplementary.pdf]

plants

MDPI

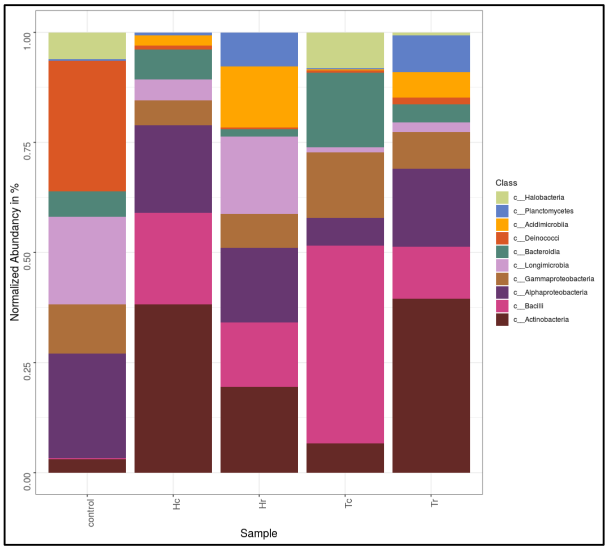

Figure S1. The abundance rates of the most prevalent class of bacterial communities in soil samples.

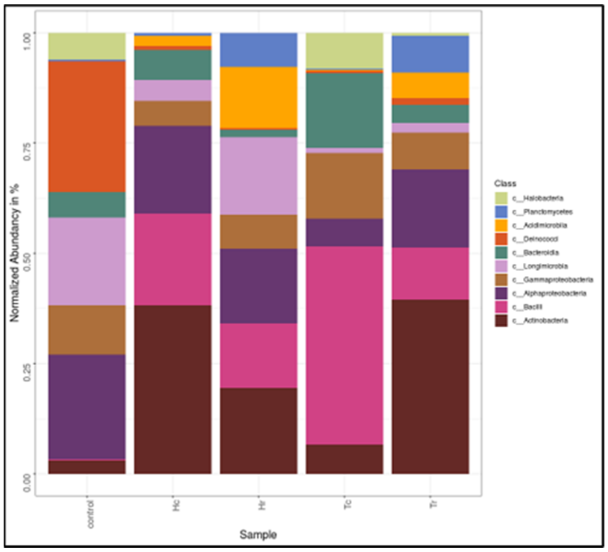

Figure S2. The abundance rates of the most prevalent order of bacterial communities in soil samples.

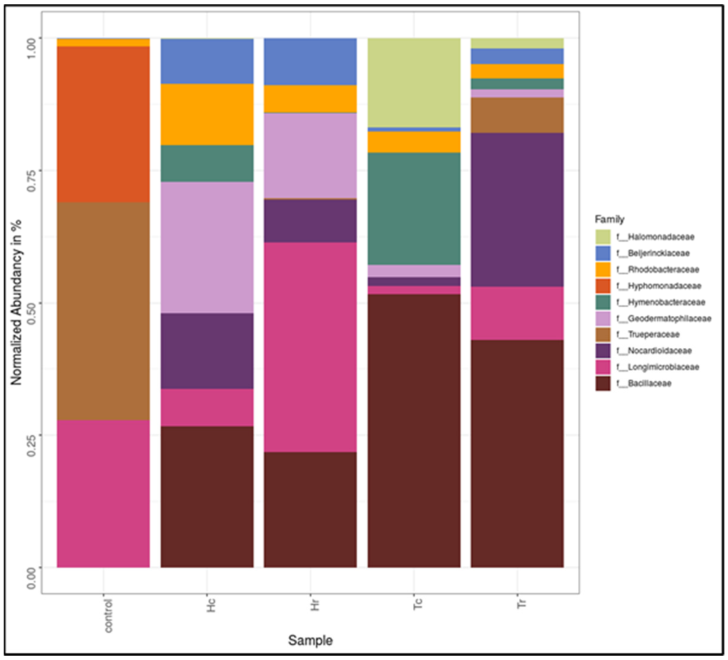

Figure S3. The abundance rates of dominant family of bacterial communities among the soil samples.

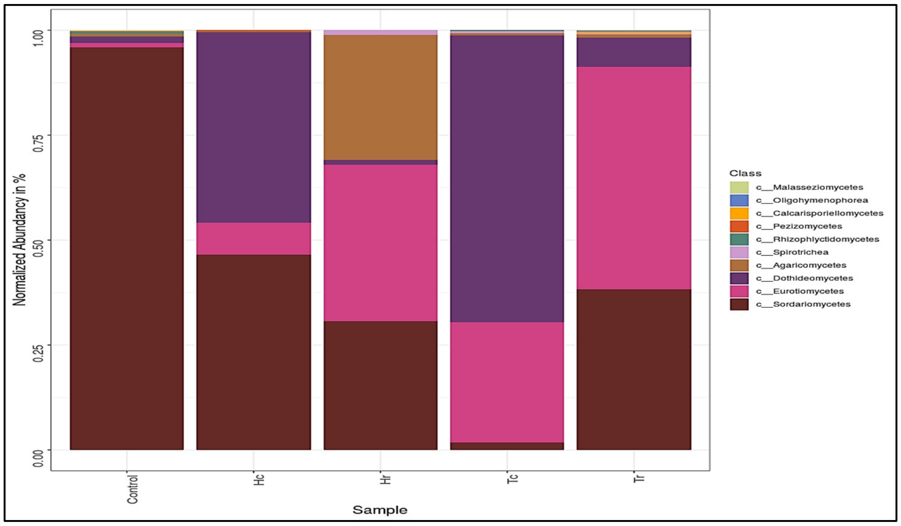

Figure S4. Fungal communities within each sample based on respective class.

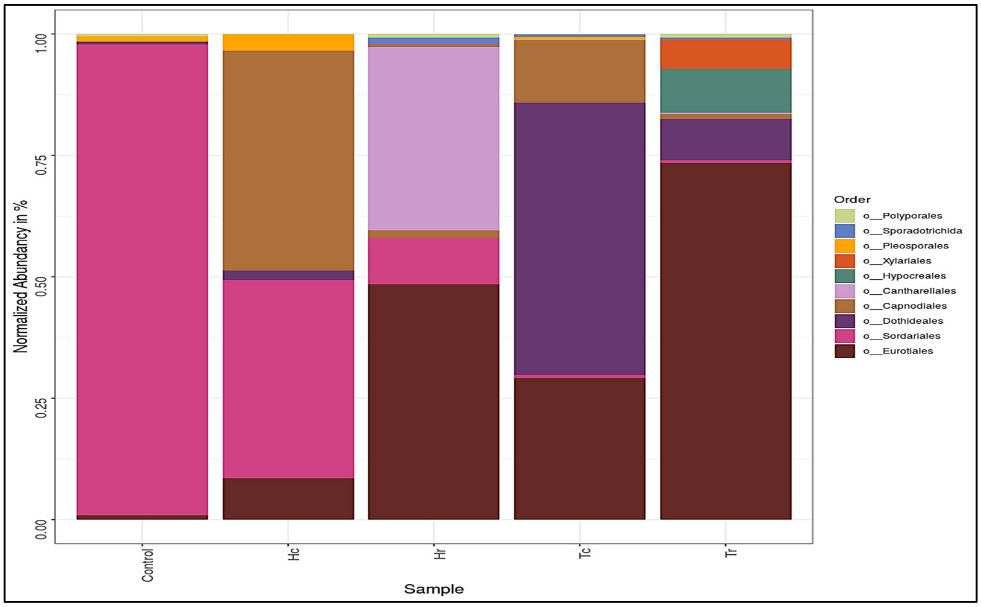

Figure S5. Fungal communities within each sample based on respective order.

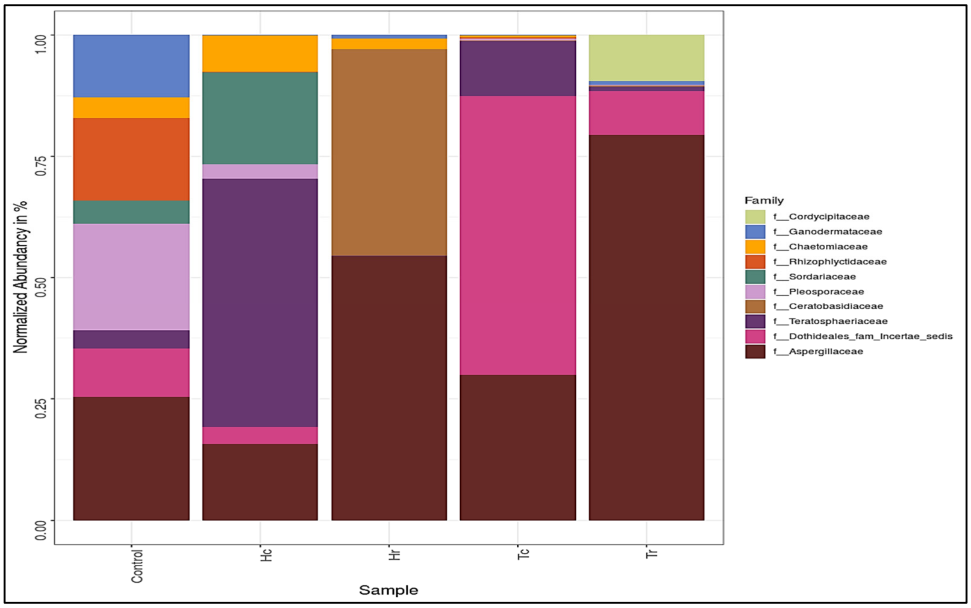

Figure S6. Fungal communities within each sample based on respective family.

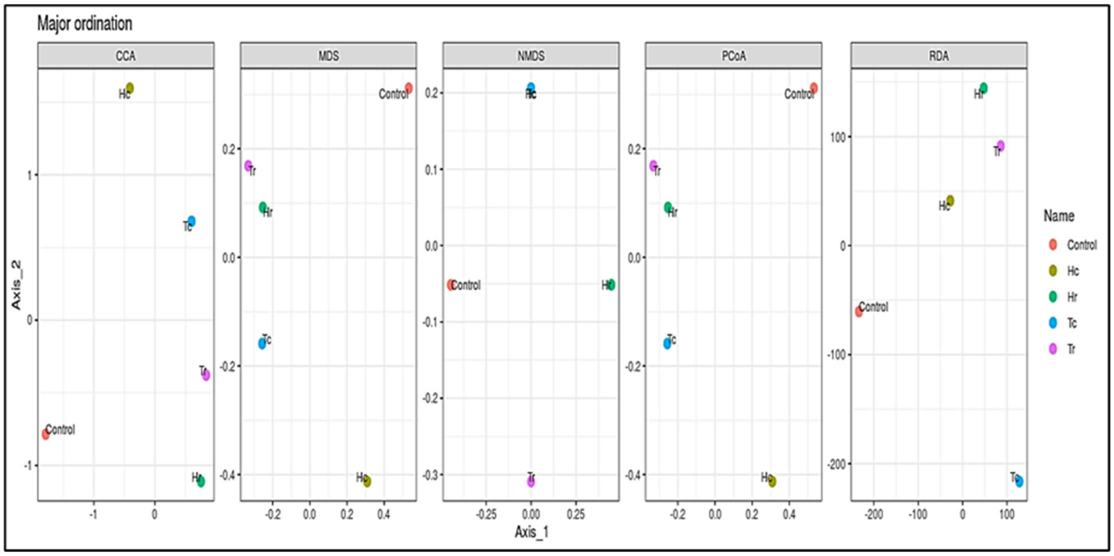

Figure S7. Box plots of beta-diversity indices of fungal community richness and composition according to CCA, MDS, NMDS, PCoA, and RDA analysis methods among the samples. Control: Control sample; (Hc): *H. perfoliata* crust sample; (Hr): *H. perfoliata* rhizosphere sample; (Tc): *T. aphylla* crust sample; (Tr): *T. aphylla* rhizosphere sample.
